# Acute Pancreatitis after Colonoscopy: A Case Presentation and Literature Review

**DOI:** 10.1155/2019/4587371

**Published:** 2019-01-15

**Authors:** Sahil D. Doshi, Yelina Alvarez, Shria Kumar, Octavia Pickett-Blakely

**Affiliations:** ^1^Perelman School of Medicine, University of Pennsylvania, Philadelphia, PA, USA; ^2^Division of Gastroenterology, Department of Medicine, Hospital of the University of Pennsylvania, University of Pennsylvania, Philadelphia, PA, USA

## Abstract

We report a case of acute pancreatitis after an elective screening colonoscopy. A 51-year-old male with a left ventricular assist device for end-stage nonischemic cardiomyopathy and a family history of colorectal cancer was admitted for an expedited heart transplant evaluation. He underwent screening colonoscopy during this admission which was technically uncomplicated apart from requiring slight maneuvering at the splenic flexure. The following day, the patient developed acute epigastric pain and one episode of emesis. Subsequent laboratory evaluation revealed a significantly elevated lipase level and cross-sectional imaging consistent with acute pancreatitis. With no evidence of gallstones, alcohol use, and hypertriglyceridemia, the acute pancreatitis was deemed to be a complication of colonoscopy. The presumed mechanism of the pancreatitis was due to mechanical trauma from insufflation and abdominal pressure, applied to at the splenic flexure, which is in close proximity to the pancreatic tail. The patient was treated with supportive care (intravenous fluid, analgesia, and pancreatic rest) and improved significantly over a three-day period.

## 1. Introduction

Colonoscopy is a common procedure for the diagnosis and management of a range of conditions and symptoms including colonic polyps, colon adenocarcinoma, colitis, and gastrointestinal (GI) bleeding. It is generally a well-tolerated and safe procedure with a low rate of serious complications. While up to 33% of patients report at least one mild, transient GI symptom, such as abdominal pain, bloating, or nausea, the rate of a serious adverse event is about 2.8 per 1000 procedures [1, 2]. These include colonic perforation (<0.1%), postpolypectomy bleeding (0.1-0.6%), and postpolypectomy syndrome (<0.2%) [1]. There are additionally less well-recognized complications including splenic rupture, acute appendicitis, and diverticulitis [2]. Acute pancreatitis is not a well-recognized complication of colonoscopy. To date, there have only been five reported cases of acute pancreatitis after colonoscopy [3–7]. We present a case report of acute pancreatitis after a screening colonoscopy.

## 2. Case Presentation

A 51-year-old male with a history of nonischemic cardiomyopathy with a left ventricular assist device was admitted for expedited heart transplant evaluation. The evaluation included an elective colonoscopy in light of a family history of colorectal cancer in his mother who died at age 61 from the disease. The patient had his first screening colonoscopy at age 45 and was diagnosed with benign polyps, which were removed, and left-sided diverticulosis. The procedure was uncomplicated and he was advised to repeat a colonoscopy in five years.

The patient was without GI symptoms at the time of his colonoscopy. He denied tobacco, alcohol, or illicit drug use. His medications included amiodarone, aspirin, famotidine, levothyroxine, lisinopril, metoprolol, sildenafil, and intravenous heparin as well as torsemide, acetaminophen, docusate sodium, and melatonin as needed. On examination, he had a left ventricular assist device port entering at the upper abdomen, but otherwise the abdomen was soft and nontender to palpation with normal bowel sounds and no appreciable masses or ascites.

The patient underwent a standard bowel preparation which included a clear liquid diet the day prior to the procedure and 20mg of Dulcolax with 4 liters of polyethylene glycol the night prior to the procedure. Monitored anesthesia care sedation was administered with propofol. The colonoscopy was performed at night without difficulty with good bowel preparation. Abdominal pressure was briefly required to maneuver around the splenic flexure. The colonoscope was advanced to the cecum with identification of the appendiceal orifice and ileocecal valve. Findings included multiple sigmoid and descending colon diverticula and two, small (<5mm) sessile polyps that were removed using cold forceps.

**Figure 1:**
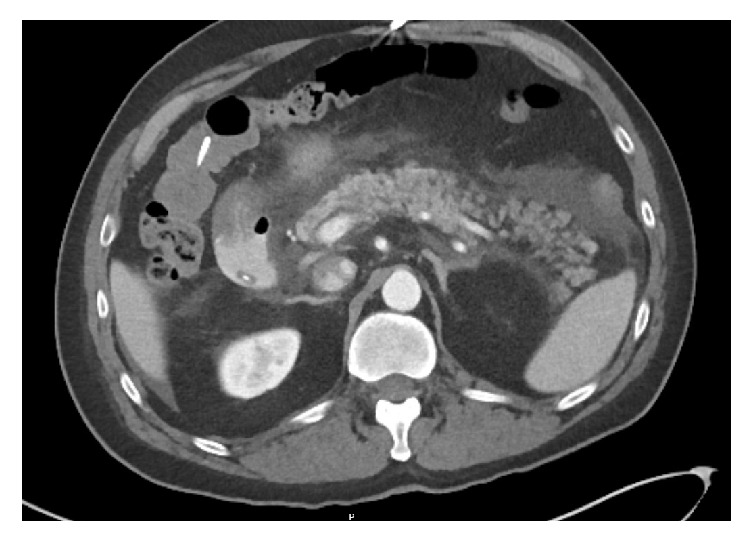
**Abdominal contrast-enhanced computed tomography image.** This image reveals diffuse edematous changes of the pancreas with surrounding inflammatory stranding and fluid consistent with acute pancreatitis.

The night of the procedure the patient had no pain or nausea and ate dinner and breakfast the following morning without incident. He then developed epigastric abdominal pain in the midmorning approximately 12-14 hours after the procedure and had one episode of nonbloody, nonbilious emesis following lunch. On physical examination, he was afebrile with a blood pressure of 104/89mmHg, heart rate of 68 beats per minute, and oxygen saturation of 100% on room air. Abdominal examination was notable for mild distension and moderate tenderness to palpation in the epigastric region without guarding or rebound tenderness and decreased bowel sounds. Laboratory examination revealed an elevated lipase of 2275 U/L and amylase of 1141 U/L. Additional abnormal laboratory findings included an elevated aspartate aminotransferase of 105 U/L, alanine aminotransferase of 94 U/L, and total bilirubin of 1.4 *μ*mol/L (normal prior to the procedure). An abdominal X-ray did not reveal an obstructive bowel gas pattern or evidence of free air. A computed tomography (CT) scan of the abdomen/pelvis revealed diffuse edematous changes of the pancreas with surrounding inflammatory stranding in the bilateral paracolic gutters, extending superiorly to the perihepatic region and inferiorly to the pelvis (Figure 1). The constellation of symptoms, labs, and imaging were suggestive of an episode of acute pancreatitis.

The patient was treated conservatively with bowel rest, intravenous fluids, and analgesics as needed. Over the next three days his symptoms and abdominal examination improved and his diet was advanced to a regular diet. The lipase normalized to 15 U/L.

## 3. Discussion

Acute pancreatitis is the leading cause of hospital admissions for GI disorders in the United States and the fifth-leading cause of in-hospital deaths [8]. The patient in this case report met diagnostic criteria for acute pancreatitis with acute epigastric pain, elevated lipase above three times the upper limit of normal, and characteristic findings on contrast-enhanced CT. The temporal nature of the colonoscopy and subsequent development of acute pancreatitis as well as the lack of significant risk factors for common etiologies of pancreatitis strongly suggest an association between the two.

Obstruction of the common bile duct by gallstones and alcohol abuse are responsible for 75% of acute pancreatitis cases [9]. The patient did not have any prior history of gallstones and although CT imaging is not best suited for biliary pathology, it did not reveal any bile duct dilation. Additionally, the patient had no history of significant alcohol use or abuse. Other etiologies include hypertriglyceridemia, hypercalcemia, anatomical anomalies, malignancy, medications, and trauma [10]. In our patient, the triglyceride level and serum calcium level were normal. There was no evidence of anatomical anomalies, such as pancreas divisum, or malignancy on imaging. The patient was not taking medications directly associated with the development of acute pancreatitis [10]. Furthermore, the patient did not experience a hypotensive episode during the procedure.

Acute pancreatitis as a complication of colonoscopy is rare: there are only five reports describing this entity to date. The reported cases and patient characteristics of acute pancreatitis following colonoscopy are summarized in Table 1. Two of these case reports specifically mention the acute pancreatitis to be localized to the tail of the pancreas [4, 6], while the remaining reports do not specify the location. In our patient, the acute pancreatitis was described to be diffuse on imaging. In these case reports, the mechanism is hypothesized to be due to mechanical injury as a result of excessive air insufflation due to difficulty passing the colonoscope at or around the splenic flexure. The splenic flexure is in close proximity to the tail of the pancreas and it is hypothesized that mechanical or barotrauma in this region (from insufflation or abdominal pressure) may precipitate acute pancreatitis. In three of the previously reported cases, the colonoscopy was noted to be technically difficult [3–5]. In one of these cases, the colonoscope initially could not be advanced beyond the splenic flexure and required withdrawal and reinsertion to successfully reach the cecum [3]. A second case also noted technical difficulty with the colonoscope unable to be advanced beyond the splenic flexure despite attempts of repositioning and application of external pressure necessitating the use of a gastroscope to advance beyond this region [4]. Similarly, in a third case, maneuvering and air insufflation at the site of the splenic flexure were required [5].

Conversely, there are cases of postcolonoscopy acute pancreatitis that did not note technical difficulty. In one report, CT imaging demonstrated hemorrhage around the tail of the pancreas in close proximity to the splenic flexure that the authors hypothesized could have been due to trauma [6], while in another report, though the colonoscopy was not technically challenging, the patient had a prior history of acute pancreatitis [7]. In our case, there was a mild degree of technical challenge negotiating the splenic flexure which required the brief use of abdominal pressure; however, the patient did not have any prior history of pancreatitis. Therefore, it was possible that minor trauma (e.g., manual pressure or barotrauma from insufflation) near the site of the splenic flexure contributed to the acute pancreatitis.

We agree with the proposed mechanism of mechanical trauma at the splenic flexure leading to pancreatic injury. Carbon dioxide is the preferred gas used for insufflation during colonoscopy in our practice and was utilized during this patient's procedure. The previously reported cases do not specify the type of gas insufflation used. Of note, our patient developed abdominal pain approximately 12-14 hours after the colonoscopy. This timeline is atypical as a few case reports have reported development of abdominal pain and acute pancreatitis within a few hours after procedure [4–6]. It is possible that our patient's pain developed earlier but that it was masked by the effects of anesthesia. Additionally, one case report does not specify exactly when the individual developed acute pancreatitis but notes that the pain developed over the days following colonoscopy [7].

It is possible that acute pancreatitis after colonoscopy, including mild episodes, is more common than reported in the literature. Abdominal pain and nausea after colonoscopy are common occurrences that occur in nearly one-third of patients and are typically benign. However, life-threatening complications such as bowel perforation are typically ruled out with diagnostic testing. The series of cases discussed here suggest that acute pancreatitis should be in the differential diagnosis in patients presenting with these symptoms in the appropriate clinical context. For example, in patients with predominantly epigastric abdominal pain or symptoms of nausea and vomiting, the diagnosis of acute pancreatitis should merit additional consideration. This is especially important as early diagnosis and management of acute pancreatitis are key to better outcomes [9].

In conclusion, we present a case of acute pancreatitis following elective colonoscopy and review the literature on previously reported cases. This complication is exceedingly rare; however, it may be more common than previously noted. It is important for endoscopists to keep this complication in mind, especially in technically challenging procedures.

## Figures and Tables

**Table 1:** Patient Characteristics of Case Reports of Acute Pancreatitis after Colonoscopy.

Study	Age	Gender	Indication	Technical Difficulty	Peak Lipase (U/L)
Khashram et al.	77	M	Change in bowel movements	Uncomplicated	4284
Ko et al.	60	F	Iron deficiency anemia	Difficult	511 (amylase)
Limb et al.	69	F	Evaluation prior to hernia repair	Uncomplicated	91 (amylase)
Shekhar et al.	25	F	Cancer surveillance	Difficult	512
Thomas et al.	25	M	Weight loss and diarrhea	Difficult	1525
Doshi et al. (this study)	51	M	Cancer screening	Uncomplicated	2275
